# Supplementary material for: Standardization of ELISA protocols for serosurveys of the SARS-CoV-2 pandemic using clinical and at-home blood sampling
Source: Nat Commun. 2021 Jan 4;12:113. doi: 10.1038/s41467-020-20383-x (PMC7782755; doi:10.1038/s41467-020-20383-x)
Supplement: Supplementary file 1 — Supplementary Information [file 41467_2020_20383_MOESM1_ESM.pdf]

SUPPLEMENTARY INFORMATION

Standardization of ELISA protocols for serosurveys of the SARS-CoV-2 pandemic using clinical and at-home blood sampling

Carleen Klumpp-Thomas*[1,2], Heather Kalish*[3], Matthew Drew[4], Sally Hunsberger[5], Kelly Snead[4], Michael P Fay[5], Jennifer Mehalko[4], Anandakumar Shunmugavel[2], Vanessa Wall[4], Peter Frank[4], John-Paul Denson[4], Min Hong[4], Gulcin Gulten[4], Simon Messing[4], Jennifer Hicks[3], Sam Michael[1], William Gillette[4], Matthew D Hall[1], Matthew Memoli[6], Dominic Esposito[4], Kaitlyn Sadtler[2†]

[1]National Center for Advancing Translational Sciences, National Institutes of Health, Rockville MD, 20850
[2]Section on Immuno-Engineering, National Institute of Biomedical Imaging and Bioengineering, National Institutes of Health, Bethesda MD 20894
[3]Trans-NIH Shared Resource on Biomedical Engineering and Physical Science, National Institute of Biomedical Imaging and Bioengineering, National Institutes of Health, Bethesda MD 20894
[4]Protein Expression Laboratory, NCI RAS Initiative, Frederick National Laboratory for Cancer Research, Frederick, MD 21702.
[5]Biostatistics Research Branch, National Institute of Allergy and Infectious Diseases, National Institutes of Health, Bethesda, MD 20894
[6]LID Clinical Studies Unit, Laboratory of Infectious Diseases, Division of Intramural Research, National Institute of Allergy and Infectious Diseases, National Institutes of Health, Bethesda, MD 20894

*these authors contributed equally to this work
[†]address correspondence to: kaitlyn.sadtler@nih.gov

TABLE OF CONTENTS:

Supplementary Figures 1 – 14
Supplementary Table 1

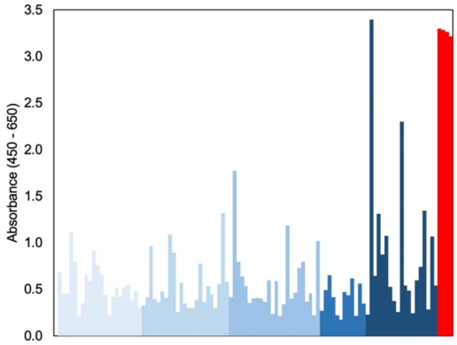

Supplementary Figure 1: Archival negative control samples at 1:100 dilution. High background observed at 1:100 dilution of sera into ELISA. Source data are provided as a Source Data file.

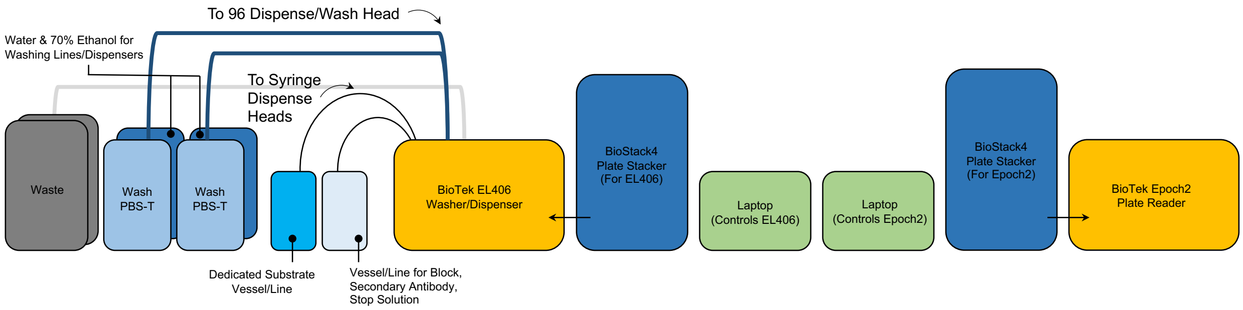

Supplementary Figure 2: Physical setup of semi-automated ELISA platform.

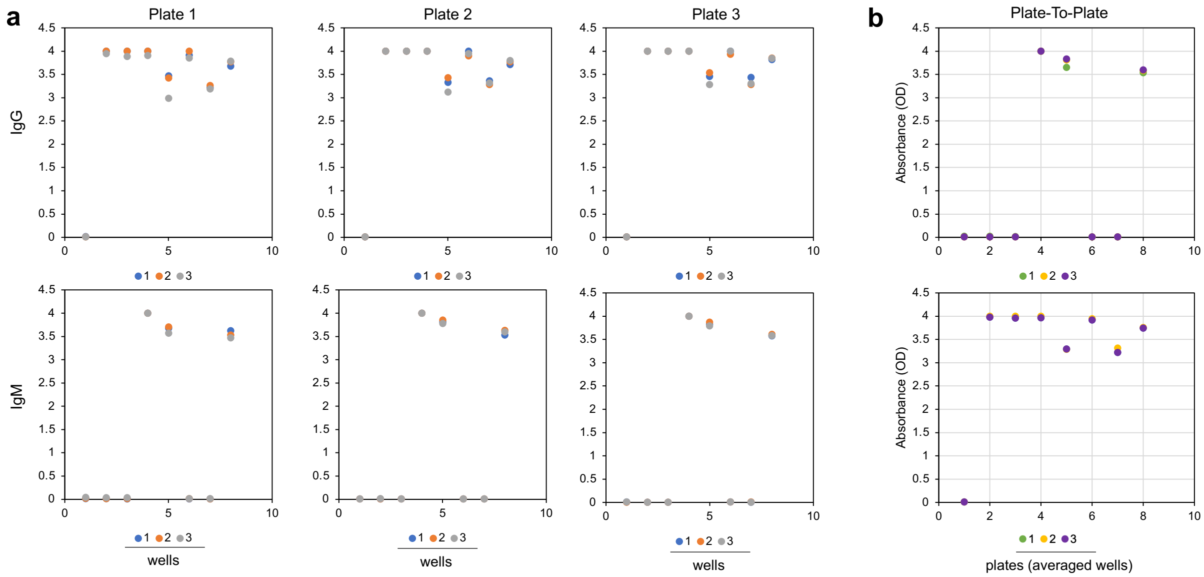

Supplementary Figure 3: Technical repeatability from well-to-well and plate-to-plate in IgG and IgM ELISA using a semi-automated setup, analyzing archival negative and convalescent positive control samples. Source data are provided as a Source Data file.

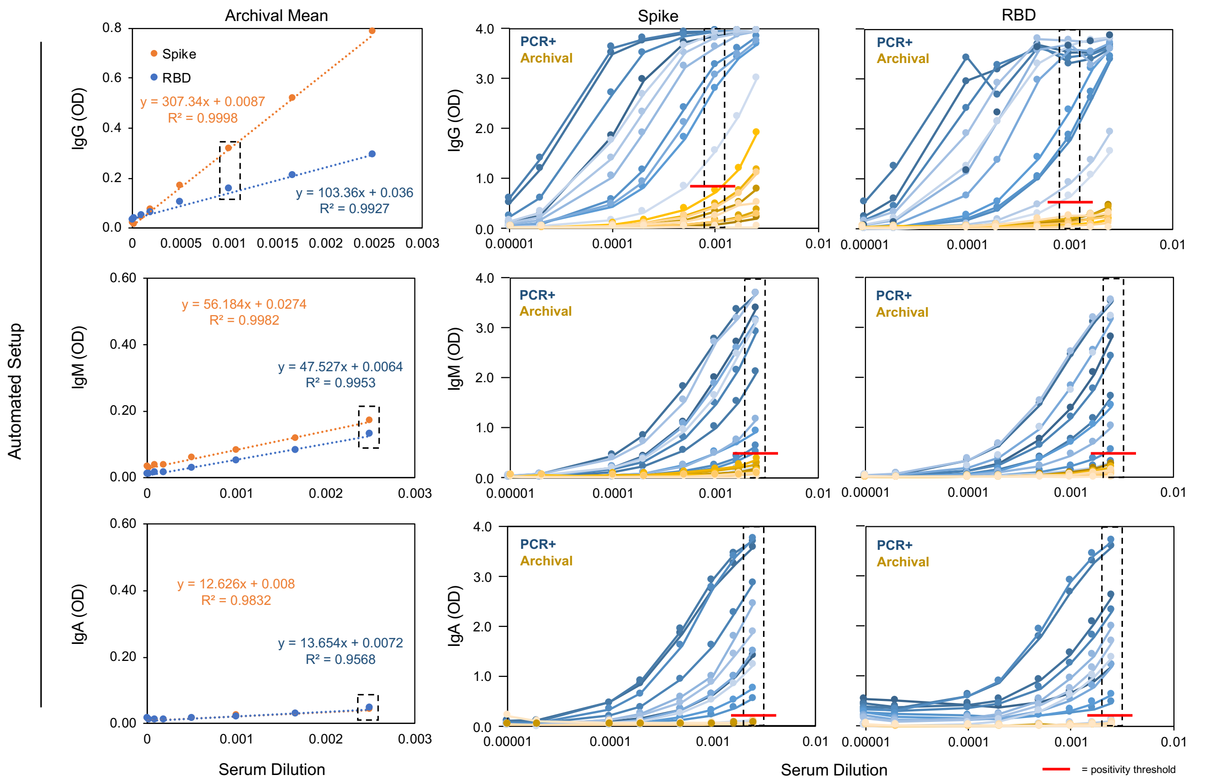

Supplementary Figure 4: Evaluation of optimal titer for automated setup. (a) Average (n = 8) archival serum signal intensity at multiple dilution titers. Dashed square surrounding lowest titer suggested to be used. (b) Titers of PCR+ confirmed patients (n = 11) for spike (left) and RBD (right). Red line = threshold of lowest titer suggested to be used. Source data are provided as a Source Data file.

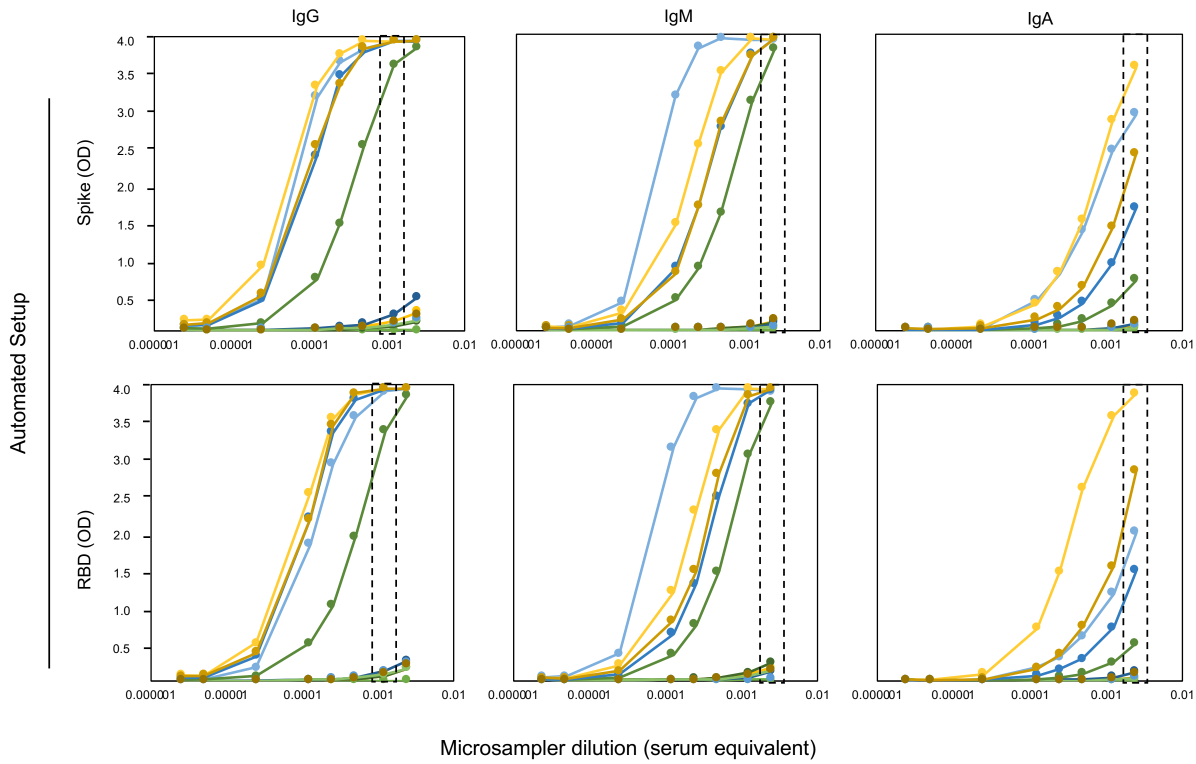

Supplementary Figure 5: Confirmation of dilution using microsamplers from high prevalence small test cohort. Titers for spike and RBD, for IgG, IgM and IgA. Square around lowest suggested titer. Titers displayed as estimate serum titer (assuming 10ul serum volume on 20ul blood microsampler and initial 1:40 dilution into elution buffer). Source data are provided as a Source Data file.

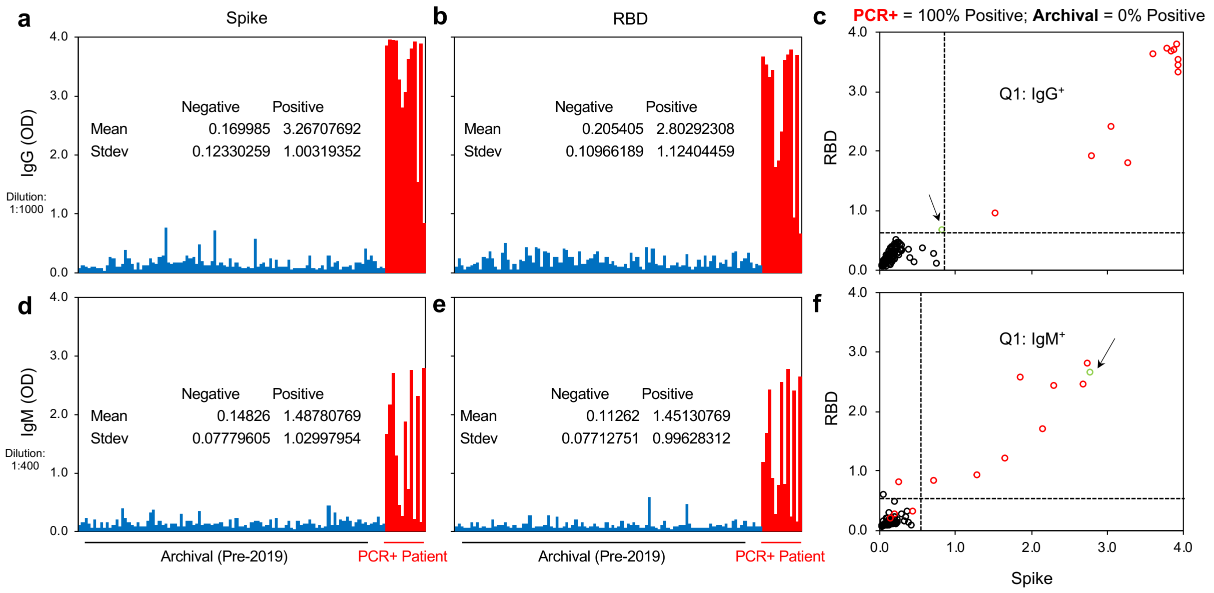

Supplementary Figure 6: Archival negative controls re-validated on semi-automated setup. (a) Spike IgG, (b) RBD IgG, (c) Threshold displayed for IgG seropositive determination. (d) Spike IgM, (e) RBD IgM, (f) Threshold displayed for IgM seropositive determination. Arrow = one PCR+ sample that displayed as IgG- but IgM+. All other PCR+ controls were IgG+ and IgM+/-. Source data are provided as a Source Data file.

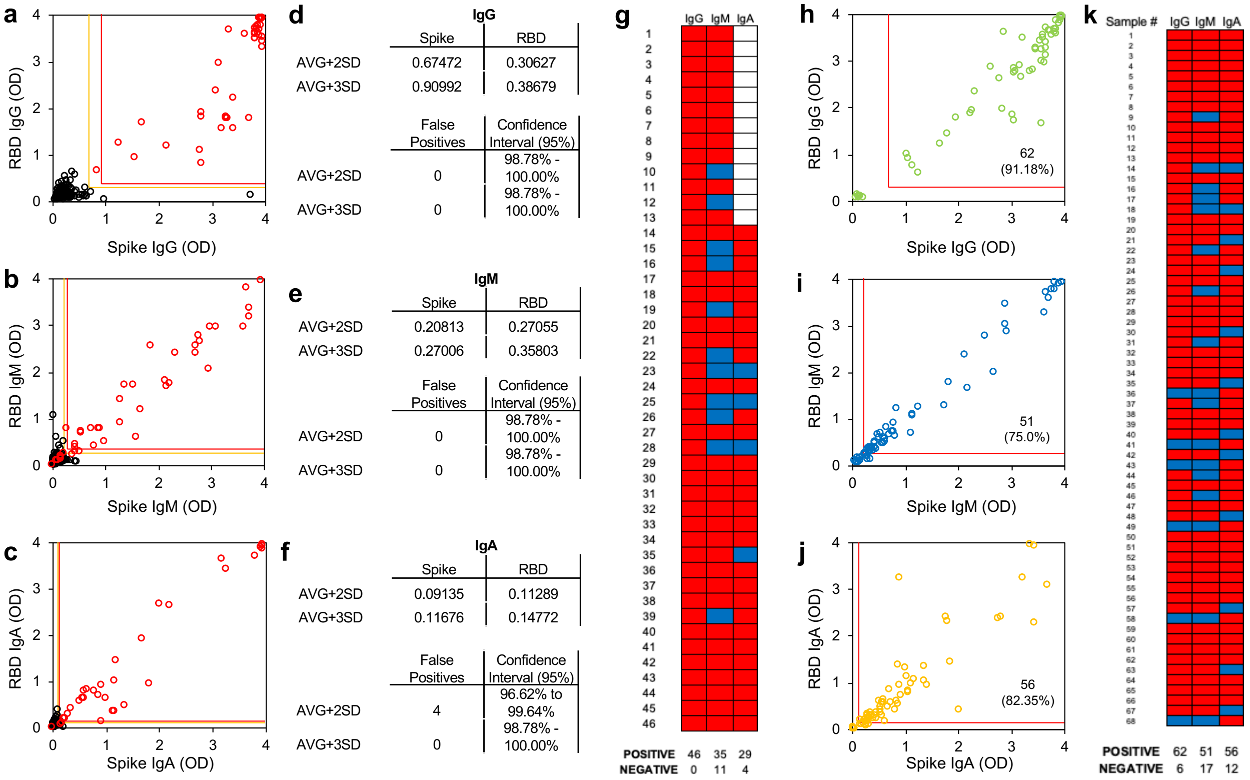

Supplementary Figure 7: Specificity of semi-automated ELISA protocol. (a-c) Absorbance (OD) of 300 archival (pre-2019) samples for spike and RBD for (a) IgG, (b) IgM, and (c) IgA. (d-f) Specificity of assay at two different thresholds: two standard deviations above the mean of the archival negative controls, and three standard deviations above the mean. (g) Sensitivity using n = 46 known seropositive controls. (h-j) Absorbance (OD) of high-incidence test cohort samples for (h) IgG, (i) IgM, (j) IgA. (k) Antibody profile of 68 donors from high incidence community. Thresholds = $\overline{x}$ + 2SD for IgG & IgM, $\overline{x}$ + 3SD for IgA. Source data are provided as a Source Data file.

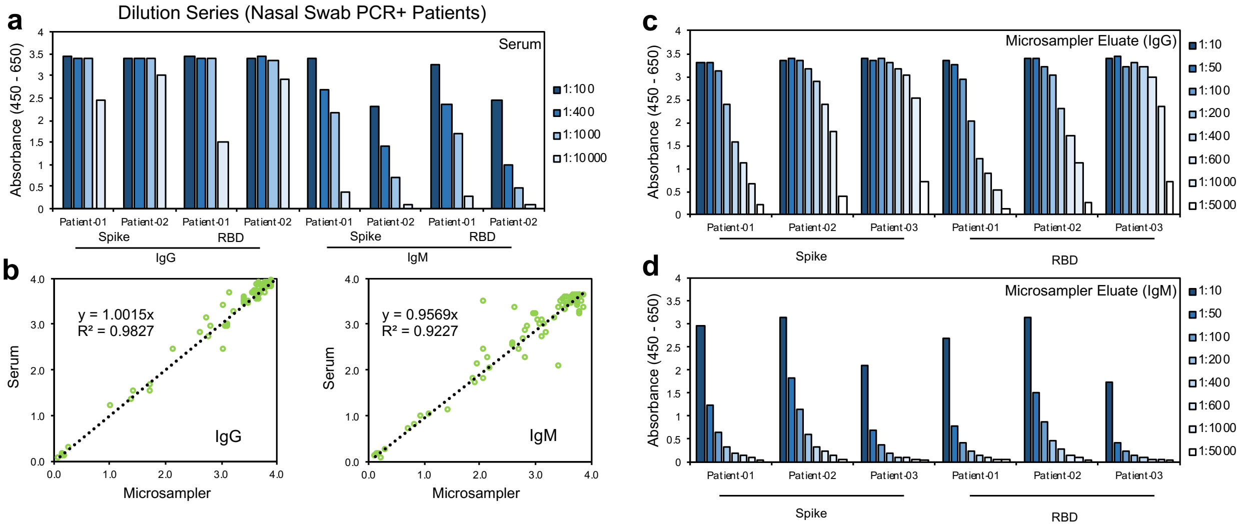

Supplementary Figure 8: Titering of serum and microsampler eluate in manual ELISA. (a) Dilution series of serum from PCR+ diagnosed patients for both IgG and IgM. (b) Linear correlation between microsampler eluate and serum from the same donor (n = 68 donors from high-exposure community, microsampler dilution = 1:10, estimated final 1:400 serum dilution equivalent based on 50% serum volume in blood, serum dilution = 1:400) (c) IgG dilution series of microsamplers from PCR+ diagnosed patients. (d) IgM dilution series of microsamplers from PCR+ diagnosed patients.

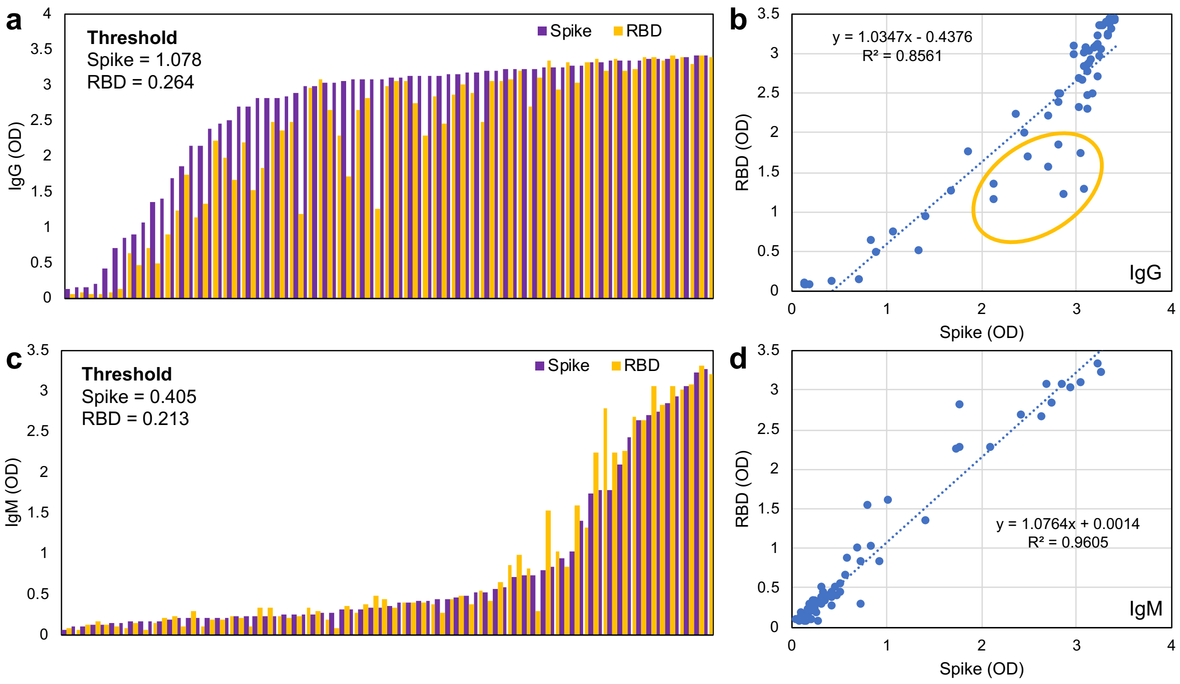

Supplementary Figure 9: Comparison of Spike and RBD signal intensity for IgG and IgM in high seroprevalence community. (**a**) IgG absorbance (OD) displayed as increasing Spike (purple) OD compared to RBD (yellow). (**b**) Correlation between Spike and RBD IgG signal intensity. (**c-d**) Spike & RBD IgM signal intensity and correlation. Source data are provided as a Source Data file.

a

$$y = a + \frac{x^m(b - a)}{x^m + c^m}$$

b

$$x = c\left(\frac{a - b}{y - b} - 1\right)^{\frac{1}{b}}$$

c

	Spike IgG	RBD IgG	Spike IgM	RBD IgM
a: Bottom	0.2265	0.1993	0.1801	0.2139
b: Top	4.025	3.988	3.856	4.006
c: IC50	0.0008215	0.0004061	0.002023	0.001046
m: Hill's Slope	2.001	1.942	1.861	2.238
	**Range at 1:400 (serum) 1:10 (microsampler eluate)**			
Threshold LOD/LOQ	0.3006	0.0656	0.1478	0.1609
Upper LOQ	159.68	92.521	574.33	116.27

Supplementary Figure 10: Sigmoidal four parameter logistic curve fitting for quantification of antibody concentrations. (a) Equation for sigmoidal 4PL where y = absorbance, a = minimum ("bottom"), b = maximum ("top"), c = IC50, m = Hill's slope, and x = antibody concentration. (b) Equation solved for x to use in calculating concentration from absorbance. (c) Variables for Spike and RBD IgG and IgM ELISAs, and quantitative range at 1:400 dilution of serum or 1:10 dilution of microsampler eluate. Threshold LOD/LOQ = limit of detection/limit of quantification calculated as the concentration at the determined threshold value for positivity. Upper LOQ = limit of quantification when the instrument reaches saturation of signal and resulting concentrations are at or above the upper LOQ. Sigmoidal 4PL calculated in GraphPad Prism.

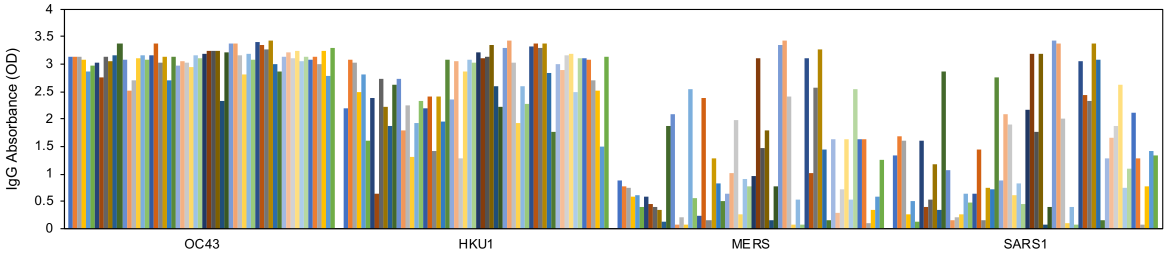

Supplementary Figure 11: Raw absorbance data of OC43, HKU1, MERS, and SARS1 for hard-hit test data set. IgG absorbance for OC43, HKU1, MERS and SARS1. Source data are provided as a Source Data file.

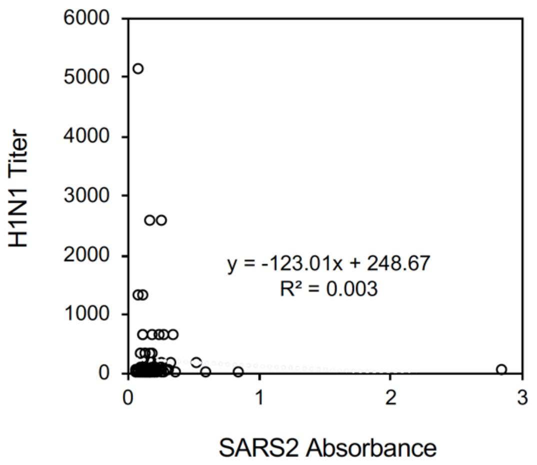

Supplementary Figure 12: H1N1 Titer as a function of SARS-2 absorbance shows no correlation between influenza titer and SARS2 signal intensity

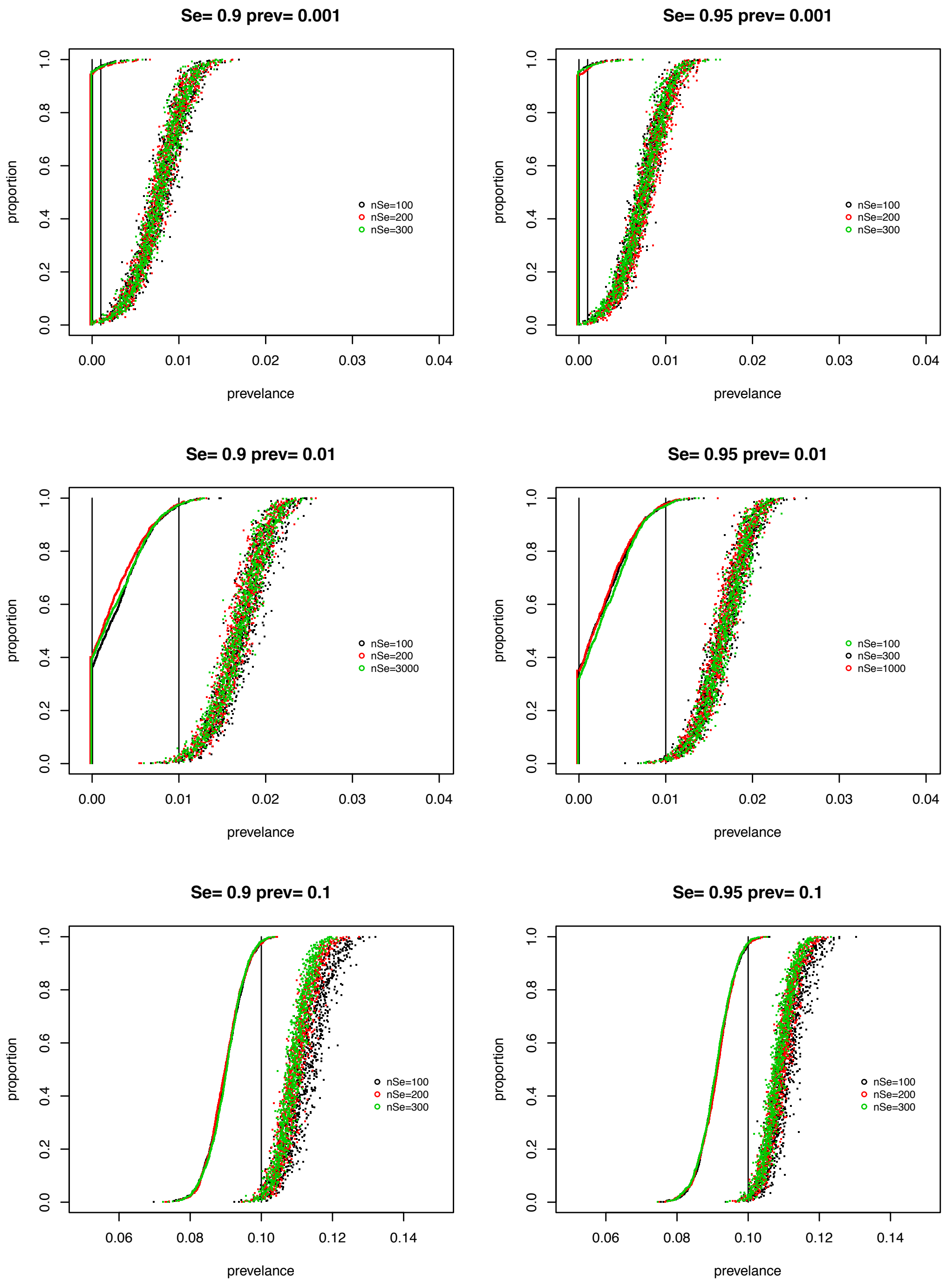

Supplementary Figure 13: Simulation and results of estimated 95% confidence intervals for serosurvey prevalence calculations. Each graph shows 95% CI's from 1000 replications of each condition. The CI's sorted by the lower bound. For graphs all graphs the true sensitivity is 0.95 and it is estimated with 1000 samples. The true specificity is either .90 or .95 and it is estimated with sample sizes of 100 (black), 300 (red), 300 (green). Each graph shows the different underlying true prevalence values of either 0.001, 0.01, or 0.1. The horizontal line shows the true prevalence for each simulation.

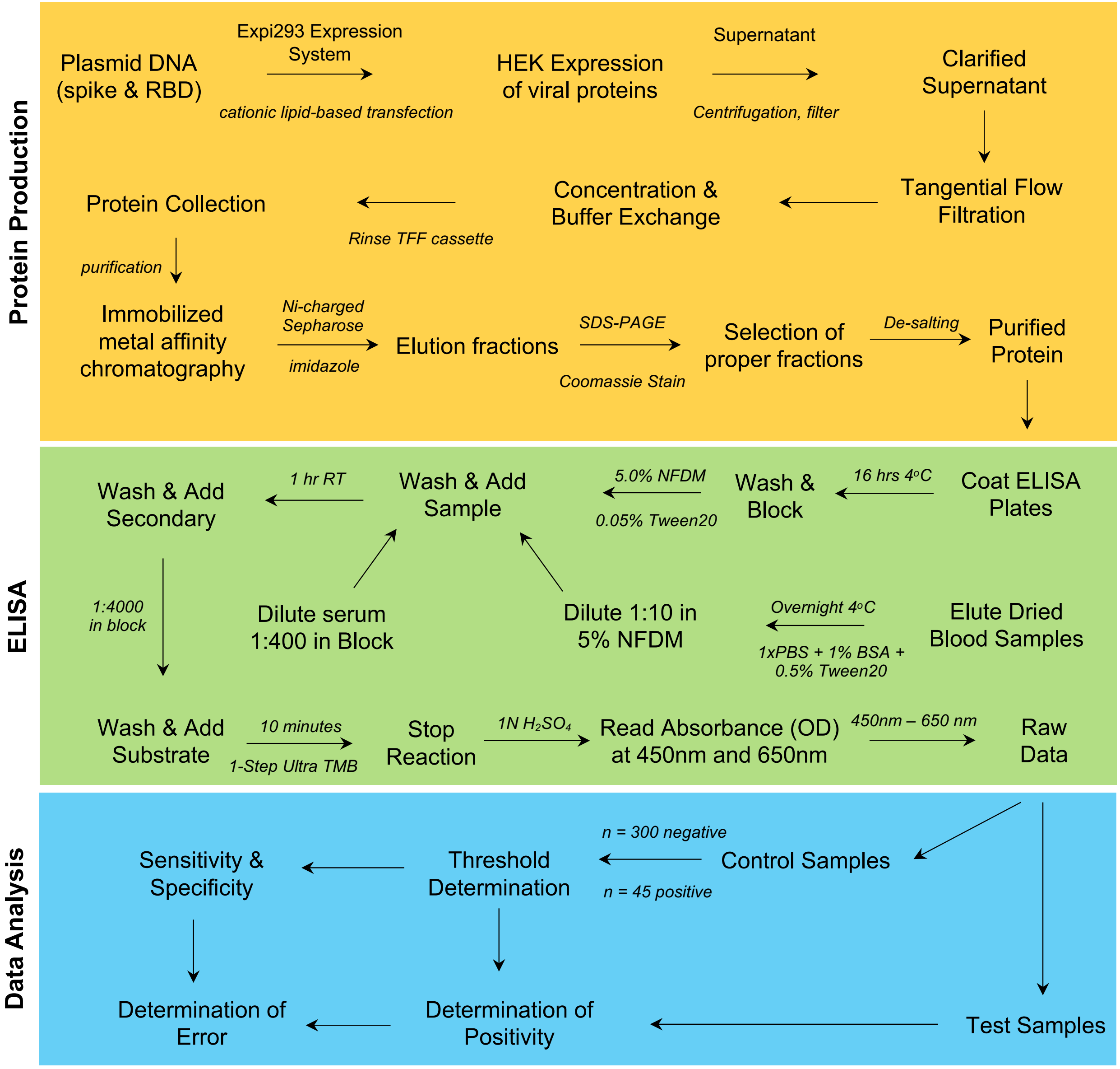

Supplementary Figure 14: Overview of the serologic assay process. This diagram reviews the overall analytic process from protein production through ELISA and data analysis.

Semi-automated High-throughput ELISA Protocol (96-well Format)

Sequence	Parameter	Value	Description
1	Reagent	100 µL	RBD or Spike protein added by multichannel pipette*
2	Time	16 hr	4ºC incubation
3	Wash	3x 300 µL	PBST using Biotek EL406 96 wash head 1st and 2nd wash aspiration height = 3.68 mm above carrier, 3rd wash aspiration height = 3.43 mm above carrier, plus secondary aspiration 2.06 mm to left of well center same height
4	Reagent	200 µL	PBST + 5.0% milk using Biotek EL406 syringe dispense head
5	Time	2 hr	RT incubation stacked with lid on top plate
6	Wash	3x 300 µL	PBST using Biotek EL406 96 wash head 1st and 2nd wash aspiration height = 3.68 mm above carrier, 3rd wash aspiration height = 4.70 mm above carrier
7	Reagent	100 µL	Samples added by multichannel pipetter after tapping out remaining wash fluid, roughly 30 mL**
8	Time	1 hr	RT incubation stacked with lid on top plate
9	Wash	3x 300 µL	PBST using Biotek EL406 96 wash head, all 3 washes at aspiration height = 3.43mm above carrier, plus secondary aspiration 2.06 mm to left of well center at same height as well as 15 sec soak post each PBST addition
10	Reagent	100 µL	Secondary antibody IgG, IgM or IgA using Biotek EL406 syringe dispense head
11	Time	1 hr	RT incubation stacked with lid on top plate
12	Wash	3x 300 µL	PBST using Biotek EL406 96 wash head 1st and 2nd wash aspiration height = 3.68 mm above carrier plus secondary aspiration 2.06 mm to left of well center at same height, 25 sec soak for each, 3rd addition of PBST left in wells, plates restacked
13	Reagent	100 µL	Substrate added using EL406 syringe dispense head after aspirating using wash head at height = 3.43 mm above carrier plus secondary aspiration 2.06 mm to left of well center at same height
14	Time	10 min	RT incubation
15	Reagent	100 µL	Stop solution added using 2nd EL406 syringe dispense head
16	Detector	Absorbance	PHERAstar FSX end-point: 450/650 (BioTek Epoch2 Tested)

* optional use of peristaltic pump on Biotek EL406 for plate coating, dead volume increases protein consumption/sample

** optional use of 96 to 96 Integra transfer device to create multiple plates from deep-well plate

Supplementary Table 1: Assay table for semi-automated ELISA for SARS-CoV-2 seropositivity evaluation. Manual ELISA tested on PHERAstar FSX, automated setup validated on both PHERAstar FSX and BioTek Epoch2 plate readers. Epoch2 saturates at OD = 4, and any overflow values are post-hoc corrected to OD = 4 for calculations.
