# Supplementary material for: Standardization of ELISA protocols for serosurveys of the SARS-CoV-2 pandemic using clinical and at-home blood sampling
Source: Nat Commun. 2021 Jan 4;12:113. doi: 10.1038/s41467-020-20383-x (PMC7782755; doi:10.1038/s41467-020-20383-x)
Supplement: Supplementary file 3 — Reporting Summary [file 41467_2020_20383_MOESM3_ESM.pdf]

Corresponding author(s): Kaitlyn Sadtler

Last updated by author(s): Nov 9, 2020

Reporting Summary

Nature Research wishes to improve the reproducibility of the work that we publish. This form provides structure for consistency and transparency in reporting. For further information on Nature Research policies, see our Editorial Policies and the Editorial Policy Checklist.

Statistics

For all statistical analyses, confirm that the following items are present in the figure legend, table legend, main text, or Methods section.

n/a	Confirmed	
☐	☒	The exact sample size (n) for each experimental group/condition, given as a discrete number and unit of measurement
☐	☒	A statement on whether measurements were taken from distinct samples or whether the same sample was measured repeatedly
☐	☒	The statistical test(s) used AND whether they are one- or two-sided<br>*Only common tests should be described solely by name; describe more complex techniques in the Methods section.*
☐	☒	A description of all covariates tested
☐	☒	A description of any assumptions or corrections, such as tests of normality and adjustment for multiple comparisons
☐	☒	A full description of the statistical parameters including central tendency (e.g. means) or other basic estimates (e.g. regression coefficient) AND variation (e.g. standard deviation) or associated estimates of uncertainty (e.g. confidence intervals)
☒	☐	For null hypothesis testing, the test statistic (e.g. F, t, r) with confidence intervals, effect sizes, degrees of freedom and P value noted<br>*Give P values as exact values whenever suitable.*
☒	☐	For Bayesian analysis, information on the choice of priors and Markov chain Monte Carlo settings
☒	☐	For hierarchical and complex designs, identification of the appropriate level for tests and full reporting of outcomes
☐	☒	Estimates of effect sizes (e.g. Cohen's d, Pearson's r), indicating how they were calculated

Our web collection on statistics for biologists contains articles on many of the points above.

Software and code

Policy information about availability of computer code

Data collection	No code was used during data collection.
Data analysis	Statistical analysis and modeling is described in detail in the Methods section.

For manuscripts utilizing custom algorithms or software that are central to the research but not yet described in published literature, software must be made available to editors and reviewers. We strongly encourage code deposition in a community repository (e.g. GitHub). See the Nature Research guidelines for submitting code & software for further information.

Data

Policy information about availability of data

All manuscripts must include a data availability statement. This statement should provide the following information, where applicable:

- Accession codes, unique identifiers, or web links for publicly available datasets
- A list of figures that have associated raw data
- A description of any restrictions on data availability

The data that support the findings of this study are available from the corresponding author upon reasonable request. The raw numbers for charts and graphs are available in the Source Data file whenever possible.

Field-specific reporting

Life sciences study design

All studies must disclose on these points even when the disclosure is negative.

Sample size	Optimization of number of controls in serologic assays was dependent upon statistical modeling displayed in the Supplemental Materials. We evalauted the number of negative controls needed for proper interrogation of seropositivity in a low incidence phenotype, and showed that n = 100 controls was not sufficient for tight confidence intervals, and n = 300 was required to tighten these intervals. Positive controls were utilized to reach a confidence interval wherein sensitivity was determined to have a lower 95% CI of >90% which was determined to be sufficient to estimate seroprevalence in a low prevalence (<10%) disease.
Data exclusions	No data has be excluded from the analysis. Positive controls were selected based on confirmation of convalescence using CLIA-certified FDA EUA serologic tests.
Replication	We repeated the assays both by manual and automated ELISA to confirm ability to repeat across platforms, and have shown plate-to-plate and technical variability in Supplemental Figure 7. Each study involved technical duplicates to quadruplicates during the study and each study was independently run two or more times.
Randomization	Archival control samples were taken randomly from a collection of pre-2019 archival controls with original serum collection distributed across months (January - December) and across multiple years (2014-2018). Test cohort samples from a high-incidence population were taken from a Hasidic Jewish community during a community blood draw in New York during April 2020, of random donors that arrived at the donation site, 68 donors were selected who reported symptoms of COVID19, 6 were selected that reported no symptoms. All samples that were collected were done at random. Technical validation studies involved repeated measurements of a lot of protein production and associated ELISA data and thus were not random as there was no manner to randomize this aspect of technical validation.
Blinding	Investigators were blinded to positive controls, and given a set of nasopharyngeal swab positive but not all convalescent samples, then results were reported back to a laboratory that measured seropositivity on EUA-granted serology assays. All samples that were positive in EUA assay were also positive in our developed assays. Data analysis was done blinded to the sample ID and completed by an individual other than the individual that ran the experimental in the laboratory.

Reporting for specific materials, systems and methods

We require information from authors about some types of materials, experimental systems and methods used in many studies. Here, indicate whether each material, system or method listed is relevant to your study. If you are not sure if a list item applies to your research, read the appropriate section before selecting a response.

Materials & experimental systems

n/a	Involved in the study	
☐	☒	Antibodies
☐	☒	Eukaryotic cell lines
☒	☐	Palaeontology and archaeology
☒	☐	Animals and other organisms
☐	☒	Human research participants
☐	☒	Clinical data
☒	☐	Dual use research of concern

Methods

n/a	Involved in the study	
☒	☐	ChIP-seq
☒	☐	Flow cytometry
☒	☐	MRI-based neuroimaging

Antibodies

Antibodies used	HRP conjugated: Goat anti-Human IgG (H+L) Cross-Adsorbed Secondary Antibody (Catalog: A18811), Goat anti-Human IgM Cross-Adsorbed Secondary Antibody (Catalog: A18835), Goat anti-Human IgA Cross-Adsorbed Secondary Antibody (Catalog: A18787); ThermoFisher. Primary anti-RBD: GenScript, human chimeric IgG (Catalog: A02038) and IgM (Catalog: A02046)
Validation	Secondary antibodies were validated against panels of archival controls (seronegative) and secondary-only controls, "blank" ELISA controls. Primary antibodies were validated by the vendor both for structure and purity and via ELISA (see product information on vendor website).

Eukaryotic cell lines

Policy information about cell lines

Cell line source(s)	Expi293 Expression System (ThermoFisher) - HEK cells
Authentication	GMP-validated cell line used in protein production was purchased directly from ThermoFisher (COA from ThermoFisher, no in-house authentication was completed)
Mycoplasma contamination	Cell lines are STR and Myco tested by manufacturer to ensure all cells are negative for Myco and STR.

Commonly misidentified lines (See ICLAC register)	There are no commonly misidentified lines in this manuscript.

Human research participants

Policy information about studies involving human research participants

Population characteristics	Serum samples collected from well-characterized healthy volunteers in NIH study NCT01386424 prior to 2019 were obtained as negative controls for SARS-CoV-2 to define the threshold for seropositivity and evaluate specificity. These were taken from a representative random population of healthy over-18 adults. These samples were deidentified and thus we have no direct information on the covariates associated with this population.
Recruitment	Control samples were sourced from a healthy volunteer population that was previously registered with the NCT01386424 trial through the Laboratory of Infectious Diseases Clinical Studies Unit. Negative control samples were sourced randomly from a representative random population of healthy over-18 adults. Positive control samples were sourced from symptomatic SARS-CoV-2 diagnosed (via PCR and FDA EUA approved tests) patients involved in other SARS-CoV-2 phenotyping studies. These samples were randomly acquired from the Maryland area and completely de-identified and uncoded so full information regarding demographics is unknown.
Ethics oversight	All clinical trials were conducted in accordance with the provisions of the Declaration of Helsinki and Good Clinical Practice guidelines through the National Institutes of Health Institutional Review Board. All clinical trial participants signed written informed consent prior to enrollment. All other samples were IRB exempt as stated by NIH OAR IRB exemption rule 4.

Note that full information on the approval of the study protocol must also be provided in the manuscript.

Clinical data

Policy information about clinical studies

All manuscripts should comply with the ICMJEguidelines for publication of clinical research and a completedCONSORT checklist must be included with all submissions.

Clinical trial registration	Archival control samples were sourced from influenza healthy donor protocol NCT01386424
Study protocol	Protocol is available through the NIH, Dr. Matthew Memoli
Data collection	Samples were collected from 2014 - 2018 and analyzed from April through June 2020.
Outcomes	Associated serosurvey (NCT04334954) is underway and initial data and results are expected by the end of October 2020.
