# Supplementary material for: Standardization of ELISA protocols for serosurveys of the SARS-CoV-2 pandemic using clinical and at-home blood sampling
Source: Nat Commun. 2021 Jan 4;12:113. doi: 10.1038/s41467-020-20383-x (PMC7782755; doi:10.1038/s41467-020-20383-x)
Supplement: Supplementary file 4 — Description of Additional Supplementary Files [file 41467_2020_20383_MOESM4_ESM.pdf]

Description of Additional Supplementary Files

File Name: Supplementary Data 1
Description: Protein sequence data for ELISA antigen constructs
